# Determinants of Refusal of A/H1N1 Pandemic Vaccination in a High Risk Population: A Qualitative Approach

**DOI:** 10.1371/journal.pone.0034054

**Published:** 2012-04-10

**Authors:** Eugenie d'Alessandro, Dominique Hubert, Odile Launay, Laurence Bassinet, Olivier Lortholary, Yannick Jaffre, Isabelle Sermet-Gaudelus

**Affiliations:** 1 CNRS, UMI 3189, Faculté de Médecine Secteur Nord, Université de la Méditerranée, Marseille, France; 2 Centre de Ressources et de Compétence en Mucoviscidose, Hôpital Cochin, Assistance Publique–Hôpitaux de Paris, Université René Descartes, Paris, France; 3 INSERM, Centre d'Investigation Clinique BT505, Hôpital Cochin, Assistance Publique–Hôpitaux de Paris, Université René Descartes, Paris, France; 4 Centre de Ressources et de Compétence en Mucoviscidose, Hôpital InterCommunal de Créteil, Créteil, France; 5 Service d'Infectiologie, Hôpital Necker-Enfants Malades, Assistance Publique–Hôpitaux de Paris, Université René Descartes, Paris, France; 6 Centre de Ressources et de Compétence en Mucoviscidose, Service de PneumoPédiatrie, Hôpital Necker-Enfants Malades, Assistance Publique–Hôpitaux de Paris, Université René Descartes, Paris, France; 7 INSERM U 845, Paris, France; University of Liverpool, United Kingdom

## Abstract

**Background:**

Our study analyses the main determinants of refusal or acceptance of the 2009 A/H1N1 vaccine in patients with cystic fibrosis, a high-risk population for severe flu infection, usually very compliant for seasonal flu vaccine.

**Methodology/Principal Findings:**

We conducted a qualitative study based on semi-structured interviews in 3 cystic fibrosis referral centres in Paris, France. The study included 42 patients with cystic fibrosis: 24 who refused the vaccine and 18 who were vaccinated. The two groups differed quite substantially in their perceptions of vaccine- and disease-related risks. Those who refused the vaccine were motivated mainly by the fears it aroused and did not explicitly consider the 2009 A/H1N1 flu a potentially severe disease. People who were vaccinated explained their choice, first and foremost, as intended to prevent the flu's potential consequences on respiratory cystic fibrosis disease. Moreover, they considered vaccination to be an indirect collective prevention tool. Patients who refused the vaccine mentioned multiple, contradictory information sources and did not appear to consider the recommendation of their local health care provider as predominant. On the contrary, those who were vaccinated stated that they had based their decision solely on the clear and unequivocal advice of their health care provider.

**Conclusions/Significance:**

These results of our survey led us to formulate three main recommendations for improving adhesion to new pandemic vaccines. (1) it appears necessary to reinforce patient education about the disease and its specific risks, but also general population information about community immunity. (2) it is essential to disseminate a clear and effective message about the safety of novel vaccines. (3) this message should be conveyed by local health care providers, who should be involved in implementing immunization.

## Introduction

Vaccines are one of the most effective tools for preventing infectious diseases, and high immunization coverage has led to drastic declines in vaccine-preventable diseases. Nonetheless, concern about adverse events associated with vaccines has risen recently in the general population, resulting in an increase in the number of people refusing vaccines and therefore the potential resurgence of these diseases [1]. Recent events, specifically the 2009 A/H1N1 influenza (A/H1N1 hereafter), showed that this concern is all the greater during a pandemic for which public perception of vulnerability to the emerging infectious disease is substantially counteracted by the fear that unsafe pharmaceuticals might be rushed to market during the health crisis [2]. This is further amplified by loss of public trust in the government's transparency and by the claims of anti-vaccine groups [3], [4].

H1N1 is a novel influenza A virus that resulted in one of the most widespread pandemics in recent history and a potential high rate of mortality in subgroups of patients with chronic diseases [5]. This prompted the development of vaccines against this virus. As it happens, the anti-A/H1N1 immunization strategy was a failure in most industrialized countries [6]–[10]. Health authorities were then blamed for mismanaging the preparedness efforts and for wasting public funds [11].

Before this pandemic, little was known about population attitudes towards new vaccines developed on an emergency basis for such situations. Identifying the determinants that influenced decisions is thus essential for developing effective strategies to overcome barriers to vaccination during future pandemics. Analysing this phenomenon in high-risk populations, which theoretically should have accepted the new vaccines, provides a unique opportunity to gain insight into how risk perception (of both pandemic effects and vaccination) predicts intention to be vaccinated. This information could help to improve the efficacy of future vaccination programmes.

Several studies have looked at various A/H1N1 vaccination programmes [8]–[31]. All highlight strong public concern about the safety of the new vaccines and the lower-than-anticipated severity of the disease. Most of these studies, however, were cross-sectional, before or during the pandemic, and based on quantitative approaches. As risk assessment also depends on a set of sociocultural factors that may change over time as the disease pattern changes, it is highly improbable that any pre-established list in quantitative questionnaires includes all possible reasons for vaccine decisions. A qualitative approach provides a better approach to understanding how vaccine-associated risk perceptions develop and how people construct their decisions for refusal of the A/H1N1 vaccine and new vaccines in general [32]. The efficacy of such studies can be increased by focusing on a population with a homogeneous background, for example, a shared disease, because patients have access to the same information from their care providers. Response heterogeneity and interpretation bias are thus lower.

In light of these considerations, we designed a study to analyse the reasons for refusal of A/H1N1 vaccination during the 2009 pandemic. Our experimental approach used a qualitative analysis and focused on a population at high risk of severe A/H1N1 infection: patients with cystic fibrosis (CF). Perceptions of vaccine- and disease-related risks in patients that declined and accepted A/H1N1 vaccination were studied. These results allowed us to formulate recommendations to improve vaccination rates in new pandemics and more generally to improve adhesion to new vaccines.

## Methods

### 1. Ethics statements

In November 2009, the “MucoFlu” research programme began in the Paris region. This cohort study sought to evaluate the clinical efficacy, immunogenicity, and tolerance of pandemic flu vaccination in patients with CF (Clinical Trials.gov registration number: NCT01499914). All patients followed in the CF centers of the Parisian area received information about the pandemic A/H1N1 flu, including a description of barrier measures, the main characteristics of the infection, the particular susceptibility of CF patients, the characteristics of vaccination against the A/H1N1 virus, and its benefits in the context of CF disease (Figure S1). Patients were advised to be immunized as soon as possible. After reception of this education leaflet by mail, all the patients were contacted by the CF nursing staff for enrollment in the cohort and vaccination. Written informed consent was obtained from all the patients for the MucoFlu Research program. An additional sheet was provided to inform on this specific anthropologic study. For the children, written informed consent was obtained from the parents but an information sheet was also provided to the children. The protocol was conducted in accordance with the Declaration of Helsinki and the relevant French statutes about biomedical research and was approved by the Ethics Committee (“Comité de Protection des Personnes Ile-de-France III”, Paris).

### 2. Study population

Vaccination adherence was assessed through a qualitative survey in patients enrolled in the cohort. CF patients were interviewed in June 2010, without any patient selection, during regular medical appointments at 3 specialised centres for CF in Paris (a pediatric centre, Necker Hospital; and two adult centres, Cochin Hospital and the Centre Intercommunal de Creteil). This method of recruitment from these cohorts of CF patients (300 children and 350 adults, respectively), during regularly scheduled appointments and without selection to avoid any bias, provided access to a fairly broad panel in terms of age and socio-professional category. For the children younger than 15 years, the parents were questioned because we assumed that the parents had made the immunization decision.

In all, 42 persons were interviewed: 24 had refused the A/H1N1 vaccine (refusers) (13 children aged from 6 to 18 years and 11 adults aged from 19 to 38 years), while 18 had been vaccinated (accepters) between November 2009 and January 2010 (8 children aged from 2 to 18 years and 10 adults aged from 19 to 55 years). Since the sample size should be directed by the research question, we stopped recruiting when new topics or factors stopped emerging from additional interviews, as stated in Pope et al [33]. There was no difference regarding socioeconomic characteristics between the refusers and the accepters.

### 3. Data collection

One of the author interviewed the patients for between one and two hours. All the interviews were fully recorded.

An exhaustive literature review allowed us to generate a list of the most common themes related to the public's acceptance of novel vaccines. An interview outline guide, based on this list, ensured systematic coverage of five main topics: (1) attitudes about vaccination and vaccination history, (2) perception of the risks related to the A/H1N1 vaccine and flu, (3) factors governing the choice about the vaccine, (4) personal preventive measures against the A/H1N1 flu other than the vaccine, and (5) information sources and content. These in-depth interviews were semi-structured to enable participants to talk in more detail about their decision. Preplanned questions were asked, and open-ended questions were formulated on the basis of participant responses.

The full interviews were transcribed from the recordings. The transcripts were read by two authors several times to identify and index themes and categories. All authors then participated in the analysis, which combined thematic analysis with constant comparison. The conceptual framework of grounded theory and the entangled social logic approach were used to interpret the data [34]–[37].

## Results

### 1. Vaccination history of interviewees

In October 2009, 37 of the 42 interviewees had been regularly vaccinated on an annual basis against seasonal flu (Table 1). Four (two in the group that received the A/H1N1 vaccine and two in the group that refused it) reported doing so irregularly, due either to omission or indecision. Only one (in the group that received the A/H1N1 vaccine) had never been vaccinated against seasonal flu. His mother explained that he was still too young (6 years), but she planned to have him vaccinated in the future.

**Table 1:** Vaccination status according to A/H1N1 vaccination status.

2009 H1N1 vaccine		Refusers(n = 24)	Accepters(n = 18)
*Vaccinated against seasonal influenza, 2009*	Yes	22	15
	Forgot	2	2
	Refused	0	1
*Vaccinated against pneumococci*	Yes	19	15
	Does not remember	5	3
	Refused	0	0
*Vaccinated against hepatitis B virus*	Yes	20	13
	Don't remember	3	5
	Refused	1	0
*History of reactions to vaccines*	None	22	16
	Mild	2	2
	Serious	0	0

The interviewees said that they were up to date with the other vaccinations: BCG, diphtheria/tetanus/poliomyelitis, pertussis, *H. influenzae* type b, measles/mumps/rubella, and meningococcal meningitis type C for the youngest subjects. Thirty-four had received pneumococcal vaccine, recommended for people with chronic respiratory diseases, and 33 the hepatitis B virus (HBV) vaccine; the others stated that they did not remember. Only one of those who had refused the A/H1N1 vaccine said he was opposed to the HBV vaccine due to the controversy regarding the risk of multiple sclerosis (Table 1).

Only four subjects, two in each group, reported a history of vaccine-related adverse effects (Table 1). They described local or moderate systemic reactions that they considered normal reactions to vaccines, while 38 said they had never had a reaction to any vaccine.

### 2. Perception of the risks related to the A/H1N1 vaccine and reasons for refusing it

Of the 24 persons who refused the vaccine, 22 reported that the main reason for their decision, far ahead of any other factor, was fear about it. They did not “have confidence” in the vaccine, which “scared” them. Above all, they mistrusted it because it was a new pharmaceutical product developed in emergency circumstances. They considered the “hastily” developed vaccine “untrustworthy”, not “100% safe”. The clinical trials seemed insufficient, the scientific safety data unreliable, and the available experience about side effects nonexistent. Except for Guillain-Barré syndrome and multiple sclerosis, mentioned by some, most interviewees were unclear about potential side effects. The vaccine represented a vague threat with long-term health repercussions as disturbing as they were unknown. This A/H1N1 vaccine was allegedly responsible for “strange things”, “serious repercussions on future life”, “unknown diseases”, and contained “dangerous substances”. At the extreme end of the spectrum, some people compared their fears with those aroused by other health events, such as “mad cow disease” and “genetically modified food”, and particularly the specific fear of being considered simply a “guinea pig”. The two refusers who did not mention fears related to the specific vaccine explained that they had not intentionally refused. One said that his general health contraindicated vaccination and the other that job constraints made it difficult for him to get to a vaccination centre.

We also explored the subjects' intentions in the event of another outbreak of A/H1N1 flu. Of those who refused the vaccine during the 2009 vaccination campaign, six thought they would refuse the vaccine again if another epidemic occurred, five said they would get it (they explained that they had been reassured and persuaded by their health care providers), and 12 reported that they were still undecided. In all cases, the recommendation of their health care provider will be key: “Now that some time has passed since the influenza A thing, and when we see all the to-do over nothing much… if my doctor advises me to get vaccinated, I'll get vaccinated if it's for the best”.

Among the 18 vaccinated subjects, perceptions of the risk related to this vaccine varied greatly. Twelve explained that they had no specific fear of the vaccine; some said they had not heard or did not remember hearing any information about side effects. Others explained that they thought it was just another vaccine. Still others mentioned that all vaccines can have side effects and the messages about potential toxicity caused them no particular concerns. Six patients mentioned fears of side effects and stated that those came explicitly from media messages about these issues. They added that the advice of their doctors had finally dissipated their concerns.

There was no difference according to age regarding perception of the risks related to the A/H1N1 vaccine.

### 3. Perception of the risks related to the A/H1N1 flu for cystic fibrosis and reasons for H1N1 vaccine acceptance

#### Vaccination as direct prevention

The perception of the risk related to the A/H1N1 flu varied widely in both groups, from barely worrisome to very troubling. However, there were globally inverse trends between the people who were vaccinated and those who refused to be.

The A/H1N1 flu aroused moderate concern in 16 of the 24 people who refused the vaccine. Eleven of those stated that they felt like “normal” people with respect to this flu (Figure S2). The other eight said they perceived it as worrisome but not sufficiently to convince them to accept the vaccination.

The vaccinated people all mentioned their particular vulnerability to the A/H1N1 flu and infectious diseases in general (Figure S2). Among them, 15 felt that influenza A was somewhat worrisome to very troubling. Furthermore, all explained that they had the A/H1N1 vaccine above all to protect themselves from the A/H1N1 flu. This notion of prevention convinced six of them immediately, while the 12 others relied on their doctors' recommendations.

There was no difference according to age regarding perception of the risks related to the A/H1N1 infection.

#### Vaccination as indirect collective prevention

In questioning the interviewees about the decisions that members of their family had made about the A/H1N1 vaccine, a new theme emerged: the concept of vaccination as collective or group prevention. Twelve of the 18 patients who had had the vaccine stated that their entire family had been vaccinated to protect the patient with CF against A/H1N1 flu. This action to prevent disease inside the family group was mentioned by 7 of the 10 adults vaccinated who were questioned and 5 of the 8 families of vaccinated children. Therefore, in that group, the collective immunity aspect of the vaccine was clearly a necessary measure, explicitly intended to protect others (Figure S3).

On the other hand, of the 24 persons who refused the A/H1N1 vaccine, only one said that her parents had been vaccinated to protect her and her brother, but had not allowed them to be vaccinated because of their concerns about side effects.

### 4. Personal preventive measures against the A/H1N1 flu other than the vaccine as alternatives to the A/H1N1 vaccine

#### Barrier measures

Of the persons who refused the vaccine, only 8 said they were concerned about the disease. All the patients who refused the vaccine used “preventive” barrier measures (Table 2). They described those protective measures as risk-free alternatives and possibly even more effective than the vaccine. More specifically, the measures ranged from simply increasing the frequency of hand washing to voluntary strict isolation (including withdrawing the child from school) and decreased or no use of public transportation, avoiding contact, wearing a mask in public places, etc.

**Table 2:** Measures of personal prevention.

2009 H1N1 vaccine		Refusal(n = 24)	Acceptance(n = 18)
*Barrier measures*	Moderate	9	18
	Substantial	15	0
*Vaccination against seasonal influenza 2009*	Protects against 2009 H1N1 flu	8	0
	Does not protect against 2009 H1N1 flu	16	18
*Oseltamivir*	Yes	5	1
	No	19	17
*Homeopathy*	Yes	2	0
	No	22	24

On the contrary, those vaccinated against the A/H1N1 virus reported barrier measures much less often. They maintained their usual hygiene habits with varying degrees of reinforcement, all of which were minor (increased frequency of hand washing, avoiding contact, etc.).

#### Pharmaceutical alternatives

To protect themselves from the flu pandemic, the people who refused the A/H1N1 vaccine mentioned various “pharmaceutical alternatives” such as the seasonal flu vaccine, oseltamivir and homeopathic remedies. People who had the A/H1N1 vaccine almost never mentioned such remedies (Table 2).

There was no difference according to age regarding preventive measures implementation.

### 5. Information sources and content

#### Information sources

We questioned the interviewees about their sources of information for the A/H1N1 flu and its vaccine. The vast majority of those who refused the vaccine mentioned multiple information sources, i.e., the media, people close to them (family, friends, and colleagues), and health care providers. They usually consulted several health care providers including doctors and nurses from the CF centres, as well as doctors and other medical professionals in private practice.

On the contrary, 15 of the persons who had the vaccine cited one information source only, i.e. their physician from their CF centre. More rarely, some also received information from other local health care providers (other medical staff from the CF centre or private practitioners, e.g., physicians, physiotherapists, and nurses).

None of the interviewees reported the Internet to be their main source of information about the A/H1N1 vaccine. Only four people (two in each group) explicitly indicated that they had searched for information about this subject on the Internet using keyword searches with search engines or searches on the web site of the patient association “Vaincre la mucovidose. No one consulted forums, blogs, or social networks (e.g., Facebook) about the vaccine.

#### Content of information

The information obtained was perceived as conflicting by 22 of the 24 people who refused the vaccine. They pointed first of all to the media, which gave voice to a succession of viewpoints ranging from alarmist to trivializing. The information in the media was deemed “too copious”, “too political”, or “too polemical”. At the same time, it provided nothing “proven” because the “media are biased” and they are only interested in “entertainment”. This media fog and its parade of those for and against the A/H1N1 vaccine, led the subjects in the end to feel “a bit lost”, “somewhat panicky”, or to “no longer really know what to think” (Figure S4). They stated that the answers and advice from their various health care providers had also been contradictory and tentative. More importantly, some added that their doctor did not give them any clear instructions about the vaccine, but left it up to them to decide whether or not to be vaccinated. These persons largely had the impression they were being left to their own devices by health care providers who were shirking their advisory responsibility. They often did not take kindly to this feeling of being “abandoned” and thus obliged to make a decision about the A/H1N1 vaccine on their own, often reluctantly.

Conversely, the people who were vaccinated considered the information they received from their health care providers to be clear and unequivocal. Sixteen said that the vaccine had been clearly recommended to them. Only two reported several conflicting sources and having had concerns. Similarly, those who had the vaccine had no feeling of having been left to their own devices about this decision. On the contrary, they all stated that their decisions were based on clear and unambiguous medical recommendations.

Patients in both groups observed a significant contrast between the reality of the epidemic (knowing few or no people who had actually had the A/H1N1 flu) and statements from the media and political authorities, which were perceived as overly alarmist. The media coverage of the erratic operation of the vaccination centres – ranging from rare mobs to desertion by the public – contributed to magnifying that impression of contradiction.

## Discussion

Our study demonstrates that the main causes for refusal of the vaccination for A/H1N1 were the perception of the risks linked to a new vaccine compared to those linked to this specific type of flu, which seemed benign and aroused only moderate concern in most of the patients who refused. Instead, prevention measures appeared to be reliable means to prevent infection. Information sources did not help, and sometimes even hindered vaccination acceptance, because they were perceived as contradictory and unreliable.

People who were vaccinated explained their choice, first and foremost, by the importance of prevention by vaccination, particularly because of their disease. They relied on the advice of their health practitioner. Moreover, they clearly associated the preventive aspect of the vaccine with its altruistic dimension: vaccination to protect others as well as themselves.

### 1. Strengths and limitations of the study

Our methodological position has a three-fold interest.

(1) Our study is the first to use a qualitative approach to better analyse the motives for refusal. Such a methodology, based on in-depth interviews allowing open questions during which people can comment freely, allows access to the experiential contexts of the interviewees, in which events unfold, risk perceptions develop, and practices are guided [32], [33], [36]. This deepens the analysis of risk-related perceptions and patient decision-making [38]. Moreover, the patients were recruited and the interviews conducted one by one as patients kept regularly scheduled appointments, without any patient selection that could have led to bias. We stopped when a point of data saturation was obtained for all of the topics examined, which constitutes strong proof of qualitative rigor [33], [35]. Therefore, although this study does not have the representative power of a randomised sample as in a quantitative study, its qualitative methodology provided a very comprehensive approach.

(2) We focused on one high risk disease group in one geographic region. CF was used as a model because patients are better-informed and educated about health issues than the general population. As they manage their chronic disease, they gain a true “lay expertise” [39]. Moreover, these patients, all received the same message from their CF doctors, supporting vaccination, including the fact that influenza virus infections present a major risk for them because they may exacerbate their respiratory disease [40]–[43]. Most importantly, CF patients are generally very compliant with seasonal flu vaccine, with coverage rates that exceed 80% [44]–[47].

These specific characteristics thus serve to eliminate the heterogeneity of samples taken from the general population. Moreover, analysing the motives for refusal of the pandemic A/H1N1 vaccine in this population highly aware of the dangers of A/H1N1 infection allows us to focus in more detail on the reasons specifically linked to the novelty of the vaccine. Finally, enrolment of pediatric and adult patients allowed us to conclude that parents of sick children do not behave differently than adult patients.

(3) Our interviews were conducted a few months after vaccination ended in people who had faced the reality of a pandemic. This contrasts to other studies, where subjects were asked about their future intentions just as the pandemic began [26]–[31]. While useful and scientifically legitimate, such prospective analyses involve a large degree of uncertainty, especially because their data rely primarily on statements out of context. Our data, based on real life experiences, allow us to evaluate the development of the respondents' behaviour. Moreover, patients in this study stated that their experience and the motives for their decision would determine their attitude in another H1N1 pandemic. The conclusions of this study are therefore useful to illuminate the behaviour of patients in future pandemics [8], [10]

### 2. Perceptions about A/H1N1 vaccine risks were the main reason for refusing the new vaccine

We found a marked discrepancy in the assessment of vaccine-related risks between refusers and accepters. The fear aroused by the vaccine was the main reason for refusal. Two principal explanations account for this fear of the vaccine: distrust of a new vaccine manufactured on an emergency basis and concern about its possible adverse effects. On this point, our results agree with the conclusions of studies conducted in the general population [8], [9], [17], [19], [26], [27], [29]–[31], [48]–[50]

The fear aroused by the A/H1N1 flu did not however result in uniform behaviours. The particular susceptibility to respiratory infections of people with CF and the importance of prevention of the A/H1N1 virus through vaccination were clear to the persons who were vaccinated, for they indicated it as the main reason for their decision. The patients who refused the vaccine described the A/H1N1 flu as rather essentially untroubling, and they trivialized, minimized, and even denied the notion of specific vulnerability in CF patients [45], [47], [51]. On the other hand, most of them implemented important barrier measures. This apparent contradiction suggests that A/H1N1 flu induced real worry in this group, although not expressed explicitly but this was not sufficient to convince them to be vaccinated. Clearly, the refusers shaped their decision in a risk-benefit approach between a perception that new vaccine equals lack of safety on the one hand and ignorance or denial of their high-risk status on the other. Thus our findings contrast with previous studies focused on specific high risk group, namely pregnant women [15], patients with cardiovascular diseases [18] or chronic respiratory diseases [52] that suggest a strong correlation between the perception of high risk relative to the A/H1N1 flu and the decision to be vaccinated.

### 3. Vaccination outlook: the altruistic attitude predicts adherence to a new vaccine

Examining what we might call the “vaccination outlook” of the interviewees, we did not find anti-vaccination attitudes in either group, or exclusive adherence to alternative medicine, or any history of serious vaccination reactions. On the contrary, the interviewees, including those who refused the vaccine, very largely adhered to vaccination principles and overwhelmingly follow vaccination recommendations, including for seasonal flu.

Our study therefore shows that the decision about A/H1N1 vaccine does not directly correlate with the attitude toward seasonal flu vaccination or, more generally, towards other vaccinations. These results contrast with the findings of Seale and Schwarzinger [26], [27] regarding the association between a positive attitude toward vaccination for the seasonal flu vaccine and adherence to the A/H1N1 vaccine in the general population.

Our results go even farther. “Vaccination outlook” differed in one essential point between persons who refused the A/H1N1 vaccine and those who took it. The community immunity preventive function of vaccines and the altruistic act of being vaccinated to protect others as well as oneself were dominant notions in persons who took the vaccine and practically absent in those who refused it. Therefore, our results suggest that the altruistic principle of vaccination in the general population is a factor that predicts adherence to a new vaccine. Similar results were also shown in health care workers [20].

Thus, one of the main foundations of the refusal process for new vaccines is not only mistrust of the vaccine itself but also the disconnection of vaccination from its altruistic and moral motivations of prevention. Without this “affective driver”, adherence to new vaccines is highly compromised by the fears to which they may give rise in western societies where safety concerns dominate and lead to demands for vaccines at “zero risk” [53]. This original result thus raises the question of the meaning of vaccination in our modern societies where new individual sensibilities coexist with changes in the circulation of pathogens. More specifically, these results should provoke policy debate on the role of the ethics of care in collective health [54].

### 4. Ecology of the vaccination campaign: general practitioner information and involvement is mandatory for the success of vaccine campaigns

Nearly all the persons who were vaccinated said they received clear, unequivocal and explicit information from their regular health care providers. On the other hand, those who refused the vaccine mentioned multiple sources (health professionals, media, friends and family) and very conflicting messages. They often perceived that the recommendations were dissonant, contradictory, and indecisive.

More specifically, they stated that the medical establishment was no longer the only legitimate stakeholder or speaker. The population received direct messages from various institutional players that fed a far-reaching controversy about the vaccine. This controversy and multiplicity of messages damaged the bond of trust that the interviewees said they had with their regular health care providers. Indeed, this health crisis was seized as an opportunity for the media and politicians to involve themselves in health policy. As they grabbed the centre of the stage, they delivered worrisome messages focused more on the potential risks of vaccination than on its benefits because not balanced by experience or true scientific information. Media studies and risk research confirm this finding and highlight the sensational nature of the coverage, which produced compelling news items to attract large audiences but little information useful to the public in deciding what they should do [31], [50], [55]. This competition aroused wide public distrust and therefore sapped patients' confidence in the information delivered by their practitioners [8], [31], [56].

Nor did the medical establishment offer a consensus about the indications for the A/H1N1 vaccine, as the refusers pointed out. A previous study focused on health care practitioners indeed showed that health care providers with inadequate knowledge about pandemic influenza A/H1N1 and its vaccine recommended vaccination less often than those who reported their knowledge as adequate [20]. Excluded from the action aspect of prescribing the vaccine, health care providers then in part offloaded responsibility for its advisory aspects [57], [58]. This abdication by physicians, leaving patients to their own devices, was widely cited by people who refused the vaccine.

Finally the interviews emphasized the contradiction the respondents felt between, on the one hand, the resources implemented by the health authorities (government communications and establishment of ad hoc vaccination centres), and the reality of the epidemic on the other. The alarming public health messages were not consistent with daily personal experience, which did not confirm the threat [8], [27]. This discrepancy between message and reality in the French context calls to mind the controversy over the HBV vaccine [59], [60]. National health authorities initiated universal HBV vaccination in the mid-1990s. However, the emotions generated by the claim that HBV vaccination might lead to multiple sclerosis resulted in a massive rejection of the HBV vaccine. Beyond this resemblance, the gap between the health authorities' message and reality reminds us of a larger set of health fears that have studded the recent history of Western countries, including, as some of the interviewees mentioned, the scandals about “mad cow” disease and GMO. This specific dimension of the 2009 A/H1N1 pandemic is again evidence of the national health authorities' difficulties in communicating about medical science and explaining vaccination procedures to the general population [61].

### 5. Implications

On the whole, the behaviours during the 2009 pandemic described above probably explain the low compliance rate for A/H1N1 vaccination throughout most industrialized countries and can be generalized to enable us to formulate recommendations to improve the likelihood of success of a future pandemic management plan.

Specifically, we have three main recommendations for improving adhesion to new vaccines:

(1) Patient education

It appears necessary to reinforce the education of patients about their disease and its specific risks to convey accurate information about the risk of the pandemic. This is in line with meta-analyses which have shown that perceptions of risk are an important predictor of uptake of vaccination against a variety of diseases [2]. We recommend that health authorities improve risk/benefit communication and invest in the implementation of effective tools for communicating vaccine risk/benefit ratios for future vaccination campaigns, emphasising the risks of not being vaccinated and the benefits of vaccination, and explicitly acknowledging and tackling safety concerns. As most of the refusers advocated the efficacy of other prevention methods than vaccines, a target action would be to convince these people that immunization provides more protection than barrier measures. Because this study showed that the accepters also based their decision on the collective immunity aspect of the vaccine, explicitly intended to protect others, we advocate that the message delivered should also consider the altruistic principle of vaccination. It is important to educate and engage citizens on the benefits of community immunity.

(2) Health care provider involvement

Health care professionals are not impersonal participants in individual and family illnesses, and it is essential not simply to treat episodes of illness, but to build a relationship and provide continuity of care [20]. Thus, the message about vaccines should first and foremost be conveyed by local health care providers, with whom patients have built strong relationships of trust [27], [45], [47], [62]. That message will be conveyed better if those professionals are involved in implementing the immunization [57], [58], as shown by previous studies of H3N1 pandemics that demonstrated both general practitioners' unique skills in empowering patients and translating national guidelines into public health education and patients' feelings that GPs' are best at helping and understanding them [63]. Primary health care providers should be the first point of contact in the health care system to provide better, comprehensive and continuing education during any emerging health crisis. We emphasize that the success of a mass vaccination campaign depends in large part on health care practitioners advising the general public to be vaccinated.

(3) The message about the vaccine: It also seems crucial to disseminate a clear and effective message about the safety of the vaccine in terms of manufacturing and validation processes, safety and efficacy [20], [26], [27], [49], [56]. In our modern societies where health and the precautionary principle must be read together, governments have a real obligation to communicate with the public about the decisions to be made regarding health interventions [64]. Because the media are an important source of information for the public during infectious disease outbreaks, it is important to provide it with regular and accurate information from the very beginning, thereby preventing public misconceptions and maintaining trust in the health authorities. We suggest that constant updates on infection rates and vaccine safety should be provided by health care authorities through the media to enable viewers to reach conclusions about their own level of risk hand and to develop a rational opinion on the vaccine's risks and benefits.

## Supporting Information

Figure S1 General Information about Influenza A (H1N1) for the parents of children with cystic fibrosis at Necker-Enfants Malades CF Center.
(DOC)

Figure S2 Perception of risks associated with influenza A/H1N1.
(DOC)

Figure S3 Vaccination to protect others.
(DOC)

Figure S4 Information. Multiplicity of sources and media fog.
(DOC)
