# Supplementary material for: Determinants of Refusal of A/H1N1 Pandemic Vaccination in a High Risk Population: A Qualitative Approach
Source: PLoS One. 2012 Apr 10;7(4):e34054. doi: 10.1371/journal.pone.0034054 (PMC3323624; doi:10.1371/journal.pone.0034054)
Supplement: Figure S1 — General Information about Influenza A (H1N1) for the parents of children with cystic fibrosis at Necker-Enfants Malades CF Center. (DOC) [file pone.0034054.s001.doc]

# Figure S1

# General Information about Influenza A (H1N1) for the parents of children

# with cystic fibrosis

# Necker-Enfants Malades CF Center

| **What are the symptoms of influenza A (H1N1)?** | |
| --- | --- |
| Sudden onset of the following signs:   - general signs: fever > 38.5° C, general feeling of sickness, aches, AND - respiratory signs: onset or worsening in coughing and congestion, respiratory difficulty   The diagnosis is based on evidence of the virus in a nasopharyngeal sample. | |
| **Is my child at greater risk of complications of influenza A (H1N1)?** | |
| Your child has cystic fibrosis. Chronic bronchitis thus makes his lungs weaker. He is therefore at higher risk than healthy children of complications, especially of superinfection. | |
| **Influenza is a contagious disease, and the virus can be transmitted two different ways** | |
| - **directly**: by respiratory pathways (droplets, coughing, spit) - **indirectly**: via hands and surfaces (doorknobs, paper, etc.) | |
| **Incubation period**: 1 to 5 days | **Contagiousness** 24 h before the onset of symptoms AND  for 7 days after symptoms begin |
| **How can I protect my child from influenza A (H1N1)?** | |
| By complying with elementary rules of hygiene, simple procedures that generally limit the risks of infection, for you and him. | |
| - Avoid insofar as possible any contact with a person with influenza or during the incubation period - Wear a surgical-type mask for protection if you have contact with a person with influenza or during the incubation period - Wash your hands carefully **several times a day** (at least, before and after meals, and in coming back home) for 30 seconds with soap and water and rub one's hands with a hydro-alcoholic solution. - Avoid very crowded areas (public transportation, department stores …) as much as possible, and if it is essential to go there, wear a surgical mask - Always use a paper tissue to blow your nose, cough, or spit, throw it in the garbage immediately after use, and wash or rub your hands afterwards. - In case of a scheduled visit at Necker-Enfants Malades, your child must wear a surgical mask as soon as he enters the hospital and rub his hands with a hydroalcoholic solution (available on entering the department).   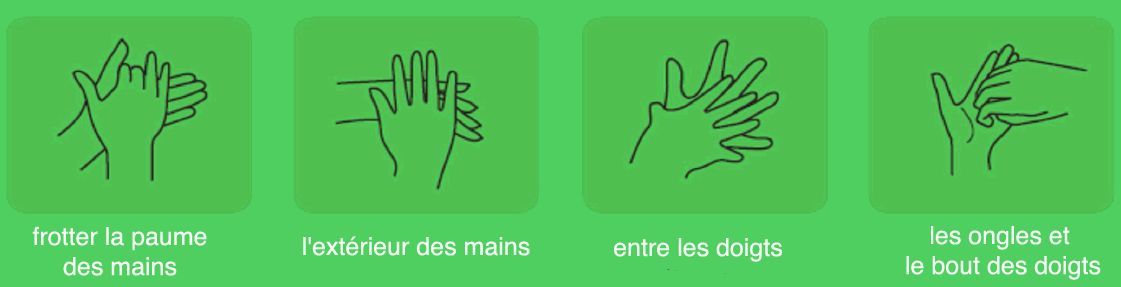  Rub your palms, the outside of your hands,  Apply the mask thorough over the entire face. Fit it over the entire face, molding it around the nose, blue side out. Do not wear the mask for more than 2 hours. Change it if it's wet.  between the fingers  and the nails and fingertips. | |

| **My child has been in close contact with someone with flu symptoms, but does not have any symptoms** |
| --- |
| **You should contact your general practitioner as soon as possible (within 48 hours at most) for a prescription to prevent** influenza, and watch for the possible appearance of symptoms (in the 5 days after the contact).  **Your child can continue to go to school.**  **If your child has appointments scheduled during this period, do not go, but notify the nurse coordinator of the CF center.** |
| **My child is a patient as Necker-Infants Malades and has flu symptoms** *(listed above)***.** |
| **A curative treatment must begin as rapidly as possible (within no more than 48 hours).**  **During the week, contact the nurse-coordinator who organizes his visits at the Cf center.**  **During the weekend, go to the nearest hospital of the Necker emergency department if you live in the Paris region. If not, call the number 15**, which will tell you where to go.  **When you come to the hospital, your child must wear a surgical mask.** |
| **Some information about influenza A (H1N1) virus** |
| This influenza A (H1N1) 2009 virus is a strain that usually affects pigs but has been infecting humans since spring 2009. Isolated first in Mexico, the virus spread rapidly across the world, triggering the first pandemic of the 21st century.  This virus is very different from those that have circulated among humans in recent years. The vaccines previously used, which contain human H1N1 strains, do not protect against this virus.  Your child must be vaccinated against the seasonal influenza as soon as the vaccine is available (see attached prescriptions). |
| **What are the possible treatments against influenza A (H1N1)?** |
| The principal drug is Tamiflu®. It is available as capsules or as a fruit-flavored liquid for small children. It can prevent the disease if it is prescribed as rapidly as possible (no more than 48 hours after exposure). It can also attenuate the symptoms and limit the risk of serious forms if it is prescribed as rapidly as possible (no more than 48 hours after the onset of symptoms). |
| **Vaccination against influenza A(H1N1)** |
| Currently, we have no information about the date that the vaccine will be available or about how it will be administered. Your child is among the groups of patients who will be vaccinated. |

Validated by the CLIN, SMIT and CRCM Necker
