# Supplementary material for: Determinants of Refusal of A/H1N1 Pandemic Vaccination in a High Risk Population: A Qualitative Approach
Source: PLoS One. 2012 Apr 10;7(4):e34054. doi: 10.1371/journal.pone.0034054 (PMC3323624; doi:10.1371/journal.pone.0034054)
Supplement: Figure S2 — Perception of risks associated with influenza A/H1N1. (DOC) [file pone.0034054.s002.doc]

**Box 1. Perception of risks associated with influenza A/H1N1**

**Perception of vulnerability in the people vaccinated**

"I have after all had cystic fibrosis since birth. Therefore, I think I needed to have the vaccine… I think there was no way that I could refuse it..."

"I had the vaccine because I have cystic fibrosis and therefore the least thing that I catch can be serious. So there were no questions to ask, I had to do it..."

**Perception of the lack of any specific risk for people who refused the A/H1N1 vaccine**

"Refusing the vaccine for my son was not a problem, because I think of him as I do of myself, I don't consider him sick … he is like any other child…"

"In relation to the vaccine, I reacted like a normal person, in good health. I didn't think about my disease, my sickness, whatever."
