# Supplementary material for: Determinants of Refusal of A/H1N1 Pandemic Vaccination in a High Risk Population: A Qualitative Approach
Source: PLoS One. 2012 Apr 10;7(4):e34054. doi: 10.1371/journal.pone.0034054 (PMC3323624; doi:10.1371/journal.pone.0034054)
Supplement: Figure S3 — Vaccination to protect others. (DOC) [file pone.0034054.s003.doc]

**Box 2. Vaccination to protect others**

"And then, the rest of the family was vaccinated… quickly, to make (her daughter with cystic fibrosis) safe … the grandfathers, the grandmothers… everyone… like an umbrella…"

"In fact, as I have two children with cystic fibrosis, I absolutely did not hesitate because I was afraid to be the flu vector; so in any case, for me, it was really that, to protect them … to screen off the children."

"So my whole family was vaccinated too… my children, even my wife… like for the (seasonal) flu vaccine, my wife does it to avoid contaminating me, giving it to me ... and then she is very protective, she doesn't want to give me something, so she didn't hesitate at all."
