# Supplementary material for: Determinants of Refusal of A/H1N1 Pandemic Vaccination in a High Risk Population: A Qualitative Approach
Source: PLoS One. 2012 Apr 10;7(4):e34054. doi: 10.1371/journal.pone.0034054 (PMC3323624; doi:10.1371/journal.pone.0034054)
Supplement: Figure S4 — Information. Multiplicity of sources and media fog. (DOC) [file pone.0034054.s004.doc]

**Box 3. Information. Multiplicity of sources and media fog.**

"When we asked, the doctors didn't know yet; we had to wait. So it was hard to get the information. And the first thing we had, it was the rumors that we heard … they came before any information, how can I say, official information, therefore, uh, we did not necessarily have good data, we heard everything and anything…"

"What I read (in the papers), it was worthless, it was … I talk a lot about politics, but it's because they were the ones who promoted that, it isn't even ... how can I say it … the doctors, quite simply. It was the politicians! I said to myself, but isn't it rather for the doctors to say? … Because doctors are generally more reassuring … "

"Uh, yes, I found that it was especially at the level of the politicians and media where that … I think from the political side, perhaps, the information wasn't expressed well… and as on the other side, there was the media who said a little of anything, uh, well, I think that people were a little lost and panicked…"
